# Biophotonic sensor for the detection of creatinine concentration in blood serum based on 1D photonic crystal

**DOI:** 10.1039/d0ra05448h

**Published:** 2020-08-27

**Authors:** Arafa H. Aly, Doaa Mohamed, Mona A. Mohaseb, N. S. Abd El-Gawaad, Y. Trabelsi

**Affiliations:** TH-PPM Group, Physics Department, Beni-Suef University Beni Suef Egypt arafa.hussien@science.bsu.edu.eg; Department of Physics, Faculty of Applied Science, Umm-Al-Qura University Mecca Saudi Arabia; Faculty of Science, King Khalid University Abha Saudi Arabia; Physics Department, College of Arts and Sciences in Muhail Asir, King Khalid University Abha Saudi Arabia; Photovoltaic and Semiconductor Materials Laboratory, National Engineering School of Tunis, University of Tunis El Manar 1002 Tunis Tunisia

## Abstract

A new biophotonic sensor based on photonic crystal (PC) has been designed for the detection of creatinine concentration in blood, and is considered an important small molecule biomarker of renal dysfunction. Based on the transfer matrix method (TMM), we theoretically investigated the transmittance spectra of a one dimensional alternating dielectric photonic crystal (PC) designed as (AB)^7^/*C*/(AB)^7^ made of MgF_2_ (A), CeO_2_ (B) and creatinine concentration present in blood (*C*). The transmission spectra exhibit resonant peaks within the photonic band gap (PBG) indicative of so-called defect modes, which depend on parameters, such as concentration of creatinine in blood, thickness of defect layer and incident angle. The proposed sensor can determine the physiological levels of creatinine in human blood serum samples. The estimated parameters realize an efficient biophotonic sensor wherein sensitivity was tuned from 136.4 nm per RIU to 306.25 nm per RIU and is very useful for the detection of creatinine.

## Abstract 2

A new biophotonic sensor based on photonic crystal (PC) has been designed for the detection of creatinine concentration in blood, and is considered an important small molecule biomarker of renal dysfunction.

## Graphical abstract

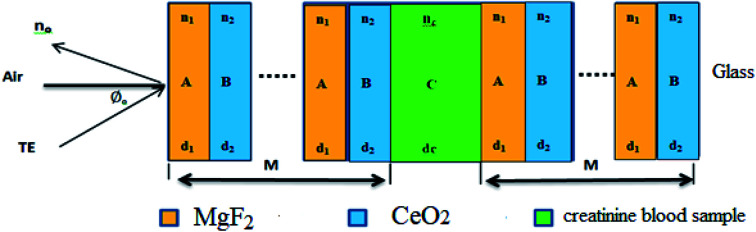

## 1. Introduction

Photonic crystals are periodic refractive index structural arrays as a function of one-dimension, two-dimension, or all three-dimension space. At the interface between every two different layers, a portion of the incident wave is reflected and due to a destructive interference between the incident wave and reflected wave, a standing wave is formed.

Photonic crystals (PCs) are structures of nanometric compositions of insulating and metal-insulating materials discovered by Yablonovitch in 1987.^1^ These structures present a modulation of refractive indices that can control the propagation of electromagnetic waves and is called the photonic band gap (PBG).^2^ The PBG consists of a range of frequencies that forbid the propagation of light and are highly sensitive to external conditions, including mechanical stress,^3^ temperature,^4,5^ electric field,^6^ magnetic field,^7^ chemicals,^8^ pressure,^9^ and self-organization of multilayered stacks.^10,11^ Therefore, PCs are considered the basis for sensing applications, such as a chemical sensors,^12^ pressure sensors,^13^ and biosensors.^14^

Amongst the sensors, biosensors are devices with mechanisms for measuring changes in biological systems. Researchers have used different sensors to detect the shift in hormones,^15^ enzymes,^16^ level of glucose in urine,^17^ cancerolic cells,^18^ and nucleic acids.^19^ Many new technologies use materials, such as hydrogels, nanoparticles and PCs for developing compact biosensing systems.^20–25^ Among them, PC-based biosensor technology is considered a simple and cost-effective method to observe various diseases much more efficiently than traditional methods.

Therefore, the considered PC technology is adequate to sense the complexity of structures of biological molecules as well as overcome the high manufacturing cost of sensors.^26–29^ Furthermore, PC-based biosensors are widely used as a diagnostic tool for assessing kidney dysfunction by measuring creatinine concentration,^30^ glucose^31–33^ and cholesterol^34^ in body fluids. There are many types of biosensors that convert signals from biological systems to electrochemical, optical, electrical or magnetic signals.

Optical sensors, in particular, play an important role in the medical field because they provide vital and complementary information.^35^ The metabolic processes in biological systems are accompanied by chemical wastes called creatinine. The creatinine is mostly responsible for the energy production in muscles. Hence, disposal of creatinine from the biological system is extremely crucial.

This operation is done by the transfer of creatinine through the blood to the kidney, which disposes it through urine. Further, the measurement of levels of creatinine in blood is considered as a strong benchmark for efficient kidney functioning.^36,37^ Therefore, the process of measuring the levels of creatinine in the blood is a very important step to identify kidney diseases.^38,39^ There are many methods to determine creatinine levels in blood and urine, such as Jaffé's method^40^ as well as enzymatic methods.^41,42^

In this study, we present a new type of biosensor based on 1D PCs that is able to estimate the levels of creatinine in blood by taking into account device factors, such as convenience, cost-efficiency of the equipment, and optimizing the time gap. Based on the TMM approach, we theoretically studied the transmission spectrum of the PC containing a creatinine sample under different concentrations. We estimated the related parameters in order to improve the sensitivity of the considered photonic biosensor.

## 2. Theoretical model

**Fig. 1:**
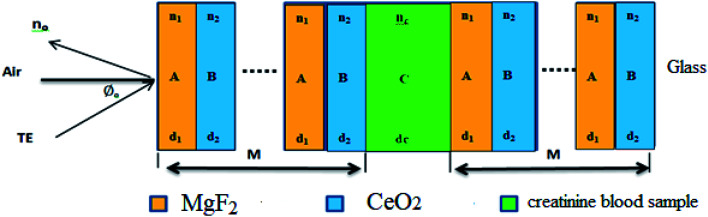
Schematic of the proposed biosensor based on 1D defective photonic crystal (PC).

We theoretically studied the transmittance spectra of a 1D defective PC designed as (AB)^*m*^/*C*/(AB)^*m*^ made up of MgF_2_ (A), CeO_2_ (B) and creatinine concentration in blood (*C*), where *m* is the number of periods. The configuration of the proposed 1D multilayered stack is shown in Fig. 1.

Based on the transfer matrix method (TMM), the interaction between stratified medium and the electromagnetic waves is described by the following equation:^43,44^1
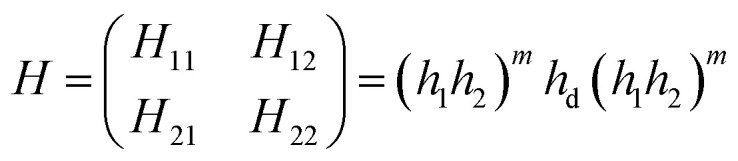
where (*H*_*ii*_)_*i*=1,2_ are the elements of the transfer matrix. Further, *h*_1_, *h*_2_, *h*_d_ are the characteristic matrix of stratified layers: MgF_2_, CeO_2_ and creatinine, respectively, and can be defined as following:^43,44^2
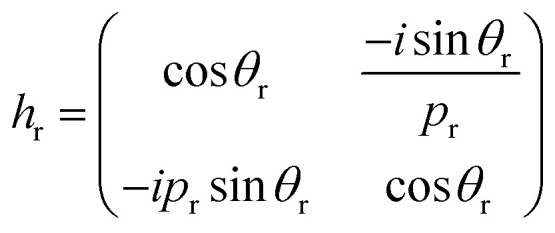
with 
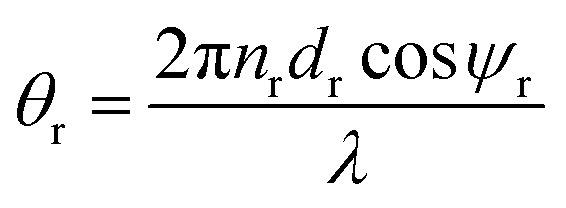
 as the phase difference at each interface. However, *p*_r_ denotes the optical thicknesses for the S-polarized wave that satisfies the equation: *P*_r_ = *n*_r_ cos *ψ*_r_, where *ψ*_1_, *ψ*_2_, *ψ*_d_ are the incident angles of corresponding materials: MgF_2_, CeO_2_ and creatinine, respectively, satisfying the Snell's law:

3 *n*_o_ sin *ψ*_o_ = *n*_1_ sin *ψ*_1_ = *n*_2_ sin *ψ*_2_ = *n*_d_ sin *ψ*_d_

The 2^nd^ kind of Chebyshev polynomials are used to calculate the matrices product (*h*_1_*h*_2_)^*m*^.^44^

Consequently, the transmittance satisfies the following expression:^45^4
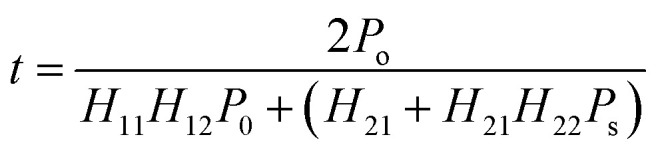
where *P*_o_ and *P*_s_ are the values of transmittance of air and substrate, respectively, and can be determined by the elements of the transfer matrix.

For the case of TE mode, the transmittance is given by the following expression:^44^5
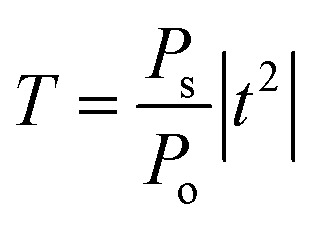


Finally, the sensitivity of the 1D-PCs can be defined as the ratio of the resonant peak wavelength to the contrast index using the following equation:^44^6
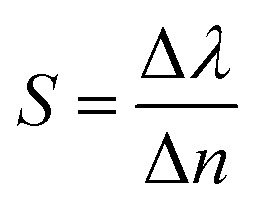
with Δ*λ* = *λ*_ab_ − *λ*_n_ and Δ*n* = *n*_ab_ − *n*_n_ are the differences of wavelength and contrast index at normal and abnormal positions, respectively.

## 3. Results and discussions

The transmittance properties of the proposed biosensor based on the 1D defective PC are investigated. The effect of relative parameters related to creatinine defects such as blood creatinine concentration, thickness and incident angle are examined in order to achieve an efficient and stable biosensor.

In the numerical calculations, the considered material parameters of multilayered stacks of layer A (MgF_2_) and layer B (CeO_2_) are the dielectrics of these materials wherein the thickness and refractive index were assumed to be *d*_A_ = *d*_B_ = 0.1 μm and *n*_A_ = 1.37 and *n*_B_ = 2.4, respectively. Further, the thickness of blood creatinine sample considered was 230 nm. The defect layer is considered in the blood creatinine sample under different concentrations.

**Fig. 2:**
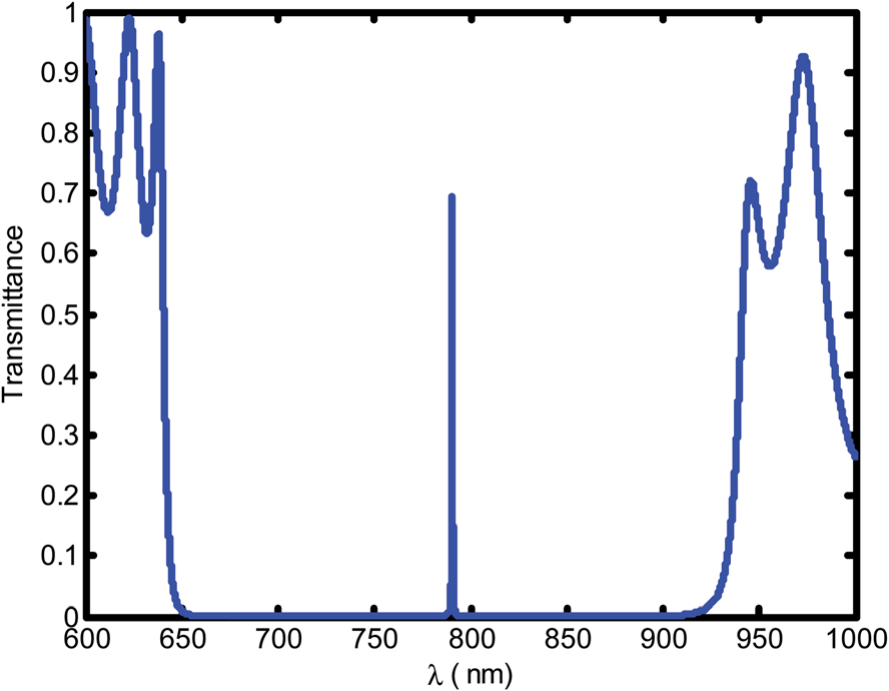
The transmittance spectrum of a defective photonic crystal as a function of wavelength at blood creatinine sample (80.9 μmol L^−1^).

Fig. 2 shows the transmittance spectra as a function of wavelengths through the defective 1D-PC at a blood creatinine concentration of 80.9 μmol L^−1^. It is obvious that a resonant transmittance peak called defect mode within the enhancement of photonic band gap (PBG) for the seventh order of periodicity is observed. Further, the PBG prohibited the propagation of wave at a corresponding wavelength range [650–927.8 nm] and allowed only the wavelength of the defect mode.

Table 1 shows the evolution of creatinine concentration (μmol L^−1^) *versus* the refractive index. It is obvious that the creatinine concentration in the sample increases with the refractive index.

**Table 1:** Creatinine concentration (μmol L^−1^) with attributed refractive index

Refractive index	Creatinine concentration (μmol L^−1^)
2.661	80.9
2.655	81.43
2.639	82.3
2.610	83.3
2.589	84.07
2.565	85.28

**Fig. 3:**
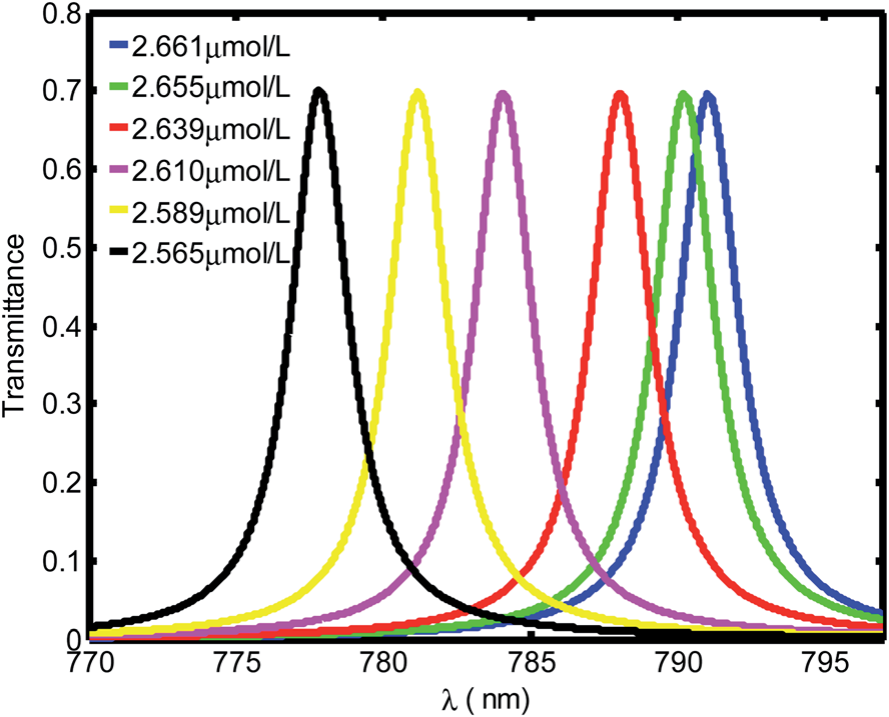
Evolution of resonant peak of defective PC as the function of wavelength with different creatinine concentrations in blood, *C*. Here, *C* is set between 2.565 μmol L^−1^ and 2.661 μmol L^−1^.

Fig. 3 shows the evolution of transmittance beam through the proposed biosensor based on defective PC at normal incidence for different blood creatinine samples, *C* (μmol L^−1^). Here, *C* is set to be between 2.565 μmol L^−1^ and 2.661 μmol L^−1^. By increasing the blood creatinine concentration, the defect mode gets shifted towards longer wavelengths. The aforementioned shift is due to the position dependent refractive index of the defect layer. Thus, this mobility is also interpreted by the condition of standing wave *Δ* = *mλ* = *n*_eff_*G*,^45^ where *Δ*, *m*, *n*_eff_ and *G* are the optical path difference, an integer, effective refractive index and geometric path difference, respectively. The proposed biosensor exhibited a sensitivity of about 136.4 nm per RIU at a normal incident angle.

**Fig. 4:**
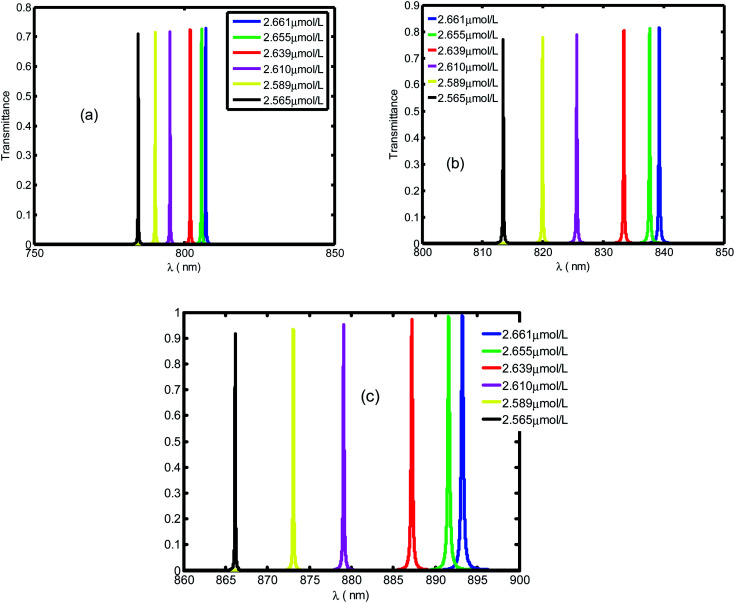
The transmittance spectrum of defective PC as a function of wavelength with different values of creatinine concentration in blood, *d*_d_. Here, the values of *d*_d_ are set at 1 μm (a), 2 μm (b) and 3 μm (c).

Fig. 4 presents the emplacement of resonant peaks within the PBG at different regions of wavelengths. We noticed that the presented defect mode shift towards longer wavelengths when we increase the thickness of defect layer. We illustrated the enhanced amplitude and number of resonant peaks for higher value of thicknesses. Thus, we obtain high sensitivity with respect to the position of defect mode as we increase the thickness. This behavior is approved experimentally.^46^ By increasing *d*_d_, the geometrical path difference of defect peaks, the sensitivity rises from 136.4 nm per RIU at *d*_d_ = 1 μm to 286.5 nm per RIU at *d*_d_ = 3 μm.

**Fig. 5:**
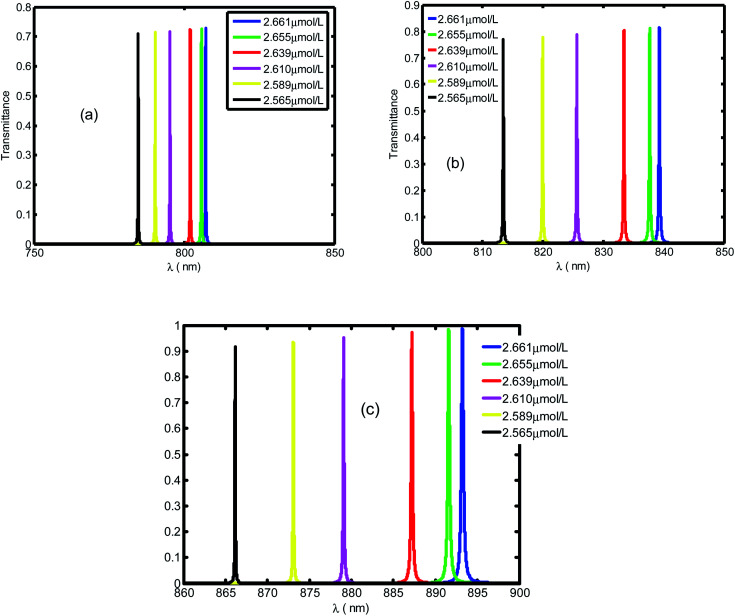
The transmittance spectrum of defective PC as the function of wavelengths with different value of creatinine concentration in blood *d*_d_. Here, *d*_d_ is set at 1 μm, 2 μm and 3 μm, respectively.

Fig. 5 displays the same behavior in Fig. 4 with resonant peaks and the defect mode shifted to longer wavelength when the defect layer thickness increased. In addition in Fig. 5 we illustrate an enhanced amplitude and number of resonant peaks for high value of thickness. Thus, we obtain an efficient sensitivity in which value increase with position of defect mode. This behavior is approved experimentally by work.^46^ By increasing *d*_d_ the geometrical path difference of defect peaks leads to rising the sensitivity from 136.4 nm per RIU at *d*_d_ = 1 μm to 286.5 nm per RIU at *d*_d_ = 3 μm.

**Fig. 6:**
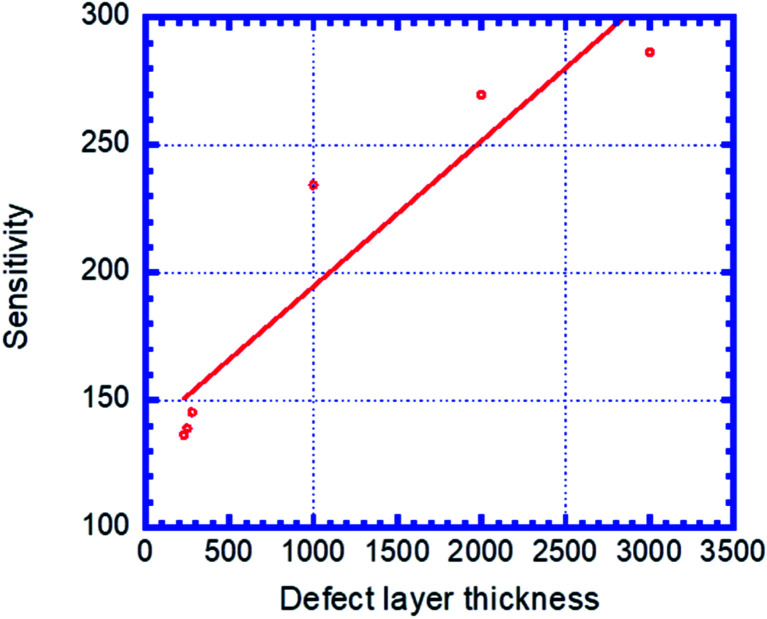
Sensitivity of the designed biosensor based on defective PC *versus* the thickness of creatinine concentration sample, *d*_d_.

Fig. 6 shows the sensitivity (nm per RIU) of the proposed biosensor based on PC *versus* the thicknesses of defect layer, *d*_d_. We see that the sensitivity increases with *d*_d_ and follows the linear fitted equation: *S* = 137.62 + 0.057069*d*_d_. Here, *R* = 0.93967 is the correlation coefficient between the linear fitting and the simulation data. *d*_d_ = 3 μm is set as the maximum thickness because an excess of this corresponding value causes the appearance of new peaks that disturb the performance of the sensor.

**Fig. 7:**
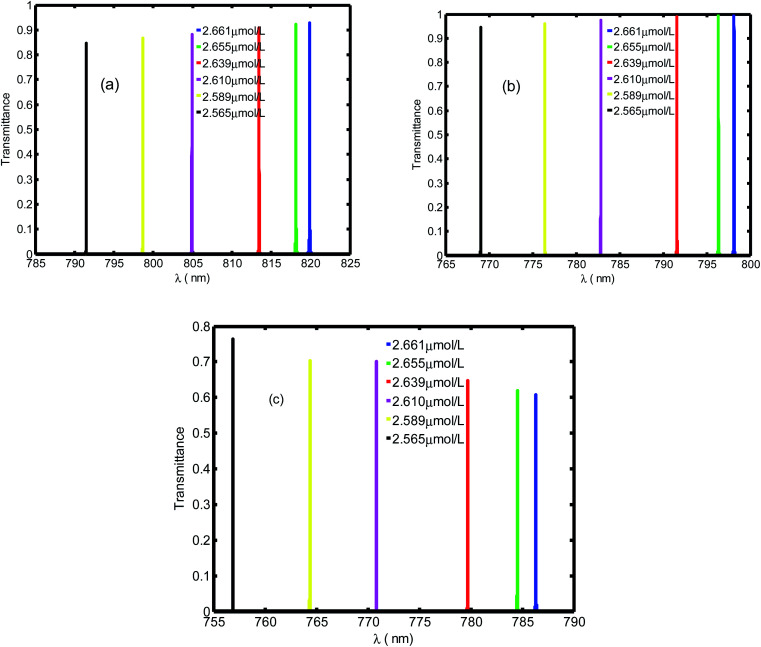
Transmittance spectra of the proposed biosensor based on defective PC at different oblique incidences with *θ* are set at (a) *θ* = 45°, (b) *θ* = 65°, and (c) *θ* = 85°.

Further, we analyzed the dependence of resonant peaks on the incident angle. For the seventh order of periodicity, we plotted the transmittance spectra as a function of the wavelength for different incident angles. Here, the incident angles are set at *θ* = 45° (Fig. 7(a)), *θ* = 65° (Fig. 7(b)), and *θ* = 85° (Fig. 7(c)). It can be seen from the Fig. 7 that with increasing angle of incidence, the defect mode shifts down to lower wavelengths, while the amplitudes of insert resonant peaks increase and become sharper as we increase the incident angle. We can explain this behavior in Fig. 7 based on Bragg Snell's law,^47^
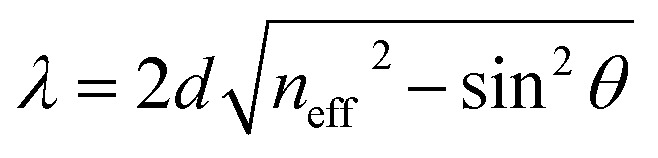
, where *m*, *λ*, *d* and *n*_eff_ are the constructive diffraction order, the wavelength of the maximum reflected intensity, the period, and the effective refractive index, respectively.

**Fig. 8:**
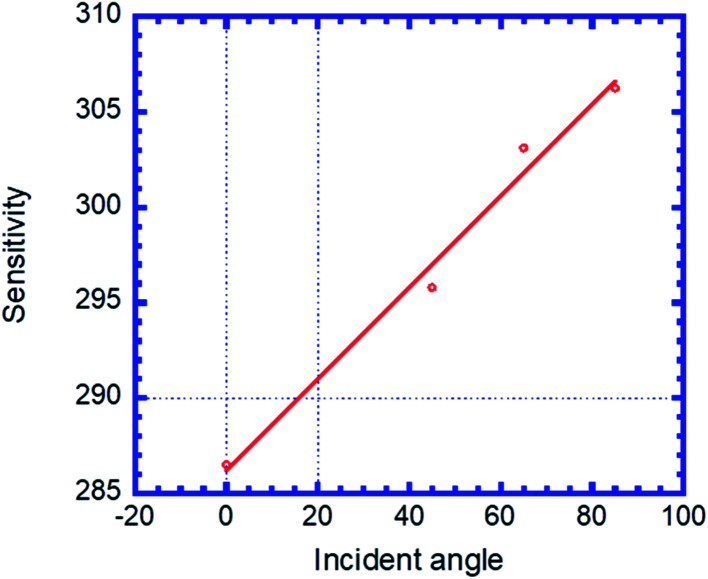
Sensitivity of the proposed biosensor based on defective PC *versus* the incident angle for the considered creatinine blood concentration.

Fig. 8 illustrates the variation in sensitivity (nm per RIU) of the proposed biosensor based on the defective PC with the angle of incidence. We see that the sensitivity (nm per RIU) increases with the angle of incidence due to the increase in amplitude of the defect mode. The linear fitted plot of sensitivity *versus* incident angle follows the equation: *S* = 286.23 + 0.23966*θ* with *R* = 0.96947, which is the correlation coefficient between the linear fitting and the simulation data.

The proposed biosensor designed (by alternating MgF_2_/CeO_2_) with creatinine sample defect exhibited an efficient sensitivity around 305 nm per RIU at high incident angles, which is not achieved for ordinary sensors, such as the theoretical optical sensor based on single nanobeam air-mode cavity (SNAC) with a sensitivity, *S* = 103 nm per RIU proposed by Yang *et al.*^48^ and the waveguide based sensor with a sensitivity, *S* = 282.4 nm per RIU proposed by Bagci and Akaoglu.^49^

To show the performance of the proposed biosensor based on the defective creatinine PC, we calculated the quality factor, 
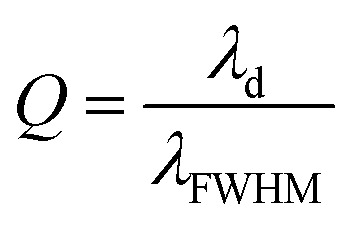
, and the ratio of sensitivity to full width at half maximum of the resonant peaks called the “figure of merit” 
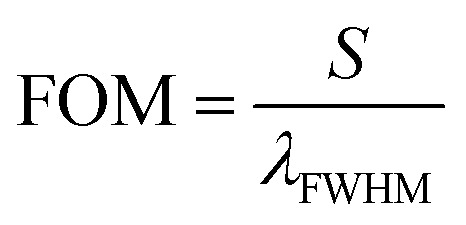
, where *λ*_d_, *λ*_FWHM_ and *S* are the resonant wavelength, the full width at half maximum (FWHM) of defect mode and sensitivity, respectively.

Table 2 shows the performance of our proposed biosensor that exhibits a high *Q*-factor about 2.5 × 10^5^ at 80.9 μmol L^−1^ and a FOM about 1.03 × 10^5^ (RIU) at 81.43 mmol L^−1^. Finally, we calculated the limit of detection (LOD) for the proposed sensor from the equation: LOD = *λ*/(20 × *S* × *Q*).^50,51^ From the simulation results in Table 2, LOD is equal 1.04 × 10^−6^ RIU. The value of LOD is very low, which means that the proposed sensor is efficient as it is capable of resolving very small changes in the refractive index.

**Table 2:** Properties of the proposed biosensor based on defective PC for different values of creatinine concentrations in blood (μmol L^−1^)

Creatinine blood sample concentration (μmol L^−1^)	*N*	*S* (nm per RIU)	*λ*_d_ (nm)	*λ*_FWHM_ (nm)	*Q* × 10^5^	FOM × 10^4^ (RIU)
80.9	2.661	—	756.9	0.003	2.5	—
81.43	2.655	306.25	764.4	0.003	2.5	10.3
82.3	2.639	306.25	770.9	0.004	1.9	7.6
83.3	2.610	306.25	779.7	0.0075	1.03	4.08
84.07	2.589	306.25	784.5	0.01	0.78	3.06
85.28	2.565	306.25	786.3	0.02	0.39	1.5

## 4. Conclusion

In this paper, a photonic biosensor based on 1D defective PC with creatinine blood sample has been developed. We theoretically investigated the performance of the proposed biosensor using the TMM approach. It is found that characteristics of the biosensor can be tuned by related parameters, such as concentration of creatinine in blood, thickness of defect layer and angle of incidence. From estimated parameters, we have obtained an efficient biosensor with high sensitivity approximating to 306.25 nm per RIU, which has not been achieved by ordinary optical sensors.

## Conflicts of interest

The authors declare they have no conflicts of interest.

## Supplementary Material
